# Methicillin-resistant *Staphylococcus aureus* acquisition in healthcare workers with cystic fibrosis: a retrospective cross-sectional study

**DOI:** 10.1186/s12890-016-0243-z

**Published:** 2016-05-11

**Authors:** Michelle E. Wood, Laura J. Sherrard, Kay A. Ramsay, Stephanie T. Yerkovich, David W. Reid, Timothy J. Kidd, Scott C. Bell

**Affiliations:** Lung Bacteria Group, QIMR Berghofer Medical Research Institute, 300 Herston Road, Brisbane, QLD 4006 Australia; Adult Cystic Fibrosis Centre, The Prince Charles Hospital, 627 Rode Road, Chermside, Brisbane, QLD 4032 Australia; School of Medicine, The University of Queensland, 288 Herston Road, Brisbane, QLD 4006 Australia; CF & Airways Microbiology Group, School of Pharmacy, Queen’s University Belfast, 97 Lisburn Road, Belfast, BT9 7BL UK; Queensland Lung Transplant Service, The Prince Charles Hospital, Brisbane, QLD 4032 Australia; Lung Inflammation and Infection Group, QIMR Berghofer Medical Research Institute, 300 Herston Road, Brisbane, QLD 4006 Australia; Centre for Experimental Medicine, The Wellcome-Wolfson Institute for Experimental Medicine, Queen’s University Belfast, 97 Lisburn Road, Belfast, BT9 7BL UK; School of Chemistry and Molecular Biosciences, The University of Queensland, 288 Herston Road, Brisbane, QLD 4072 Australia

**Keywords:** MRSA, Cystic fibrosis, Employment, Incidence, Nosocomial infection

## Abstract

**Background:**

People with cystic fibrosis (CF) may work in healthcare settings risking nosocomial pathogen acquisition. The aim of this study was to determine the incidence of methicillin-resistant *Staphylococcus aureus* (MRSA) infection in adult healthcare workers with CF (HCWcf).

**Methods:**

Data was collected in this observational study on MRSA acquisition from 405 CF patients attending an adult CF centre in Australia between 2001–2012. Demographic and clinical characteristics were compared between HCWcf and non-HCWcf. A sub-analysis was subsequently performed to compare demographic and clinical characteristics between those patients (HCWcf versus non-HCWcf) that acquired MRSA. We also investigated rates of chronic MRSA infection and the outcome of eradication treatment in HCWcf.

**Results:**

A higher proportion of HCWcf acquired MRSA [*n* = 10/21] compared to non-HCWcf [*n* = 40/255] (*P* <0.001). The odds of MRSA acquisition were 8.4 (95 % CI, 3.0 – 23.4) times greater in HCWcf than non-HCWcf. HCWcf with MRSA were older (*P* = 0.02) and had better lung function (*P* = 0.009), yet hospitalisation rates were similar compared to non-HCWcf with MRSA. Chronic MRSA infection developed in 36/50 CF patients (HCWcf, *n* = 6; non-HCWcf, *n* = 30), with eradication therapy achieved in 5/6 (83 %) HCWcf.

**Conclusions:**

The rate of MRSA incidence was highest in HCWcf and the workplace is a possible source of acquisition. Vocational guidance should include the potential for MRSA acquisition for CF patients considering healthcare professions.

## Background

Cystic fibrosis (CF) is the most common, life-limiting genetic disease in the Caucasian population. Recent studies predicted median survival of people with CF to increase beyond 50 years [1, 2]. Consequently, these individuals have the opportunity to pursue careers, including training and employment in health-related fields. A survey reported that ~7 % of adults with CF work in healthcare professions in the UK [3].

Methicillin-resistant *Staphylococcus aureus* (MRSA) is a major endemic pathogen in many hospitals posing an important source of colonisation for staff with ~5 % of healthcare workers developing non-fatal clinical MRSA infections such as of the skin or soft tissue, or life-threatening infections in *at risk* patients [4, 5]. In patients with CF, chronic airway infection with MRSA has been associated with poorer clinical outcomes [6, 7] and an increased requirement for hospitalisation and antibiotic usage [8].

The main aim of this study was to determine and compare acquisition of MRSA amongst adult healthcare workers with CF (HCWcf) and non-HCWcf. Rates of chronic MRSA infection and the outcome of eradication therapy were also investigated.

## Methods

### Study design

Of the more than 400 adults with CF in Queensland (Australia), ~70 % receive their care at The Prince Charles Hospital (TPCH), one of the largest adult CF centres in the Southern Hemisphere. This study included CF patients (≥18 years, *n* = 405), who attended the adult CF Centre, between 2001 and 2012. Ethics approval for this project was granted by The Prince Charles Hospital Human and Research Ethics Committee, Metro North Hospital and Health Service, Brisbane, Queensland, Australia (HREC/13/QPCH/51).

**Fig. 1:**
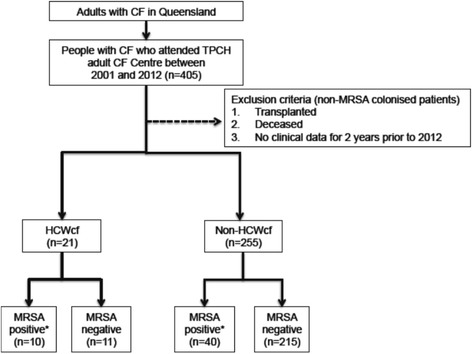
Cross-sectional observational study design. This study identified patients with CF, who were MRSA culture positive and were either a HCWcf or a non-HCWcf. *Abbreviations:* HCWcf, adult healthcare workers with CF; non-HCWcf, adult non-healthcare workers with CF; TPCH, The Prince Charles Hospital. *Multiresistant MRSA was detected in 5 HCWcf and 30 non-HCWcf. Non-multiresistant MRSA was detected in 5 HCWcf and 10 non-HCWcf

Figure 1 describes the design of this study. Briefly, patients with CF were stratified into one of two groups depending on whether they were a HCWcf or a non-HCWcf. Each group was further sub-divided into positive or negative for MRSA based on sputum culture results.

A healthcare worker was defined as a person who worked or undertook a clinical placement within a healthcare facility (e.g. hospital, pharmacy) and had frequent, direct patient contact. This group also comprised veterinarians as it is recognised that people with animal contact might have a greater chance of MRSA acquisition [9].

### MRSA acquisition

TPCH Adult CF Centre database of all respiratory microbiology was reviewed and positive MRSA infections were collated. All cases of MRSA acquisition were identified and the date of MRSA acquisition was defined as the first positive sputum culture recorded by the hospital microbiology laboratory. If a person was MRSA positive (i) at the start of the study period in 2001 (*n* = 12), or (ii) when transferred to care at TPCH (*n* = 6), the patient’s previous hospital records were reviewed to determine the date of acquisition. All patients included in this study acquired MRSA between 1998 and 2012.

### Clinical data

Clinical data were collected from medical records and sputum microbiology was determined from the TPCH CF database, where available. Demographics and clinical parameters (i.e. age, gender, forced expiratory volume in one second percent [FEV_1_%] predicted, pancreatic sufficiency status, hospitalisation days and admissions and details of *Pseudomonas aeruginosa* (*P. aeruginosa*) infection were determined for the entire cohort. Data for MRSA positive patients corresponded to the date of initial acquisition. In the non-MRSA group, data was collected at 2012 and excluded those who had been transplanted, were deceased or had not been a clinic patient for the full 2 years prior to 2012 (Fig. 1).

Results of peripheral skin screening swabs (nose, axilla and groin) collected subsequent to MRSA respiratory acquisition were reviewed, with individuals recorded as either positive or negative for peripheral MRSA colonisation.

### Classification of MRSA and eradication therapy

At the time of data collection, microbiology laboratories in Queensland differentiated isolates phenotypically as multiresistant MRSA (mMRSA) or non-mMRSA (nmMRSA) strains. Briefly, mMRSA was identified if an isolate was resistant to all β-lactam antibiotics and to three or more non-β-lactam agents (e.g. clindamycin, tetracycline, rifampicin). An isolate was defined as nmMRSA if resistance to all β-lactam agents was apparent but susceptibility to two or more non-β-lactam antibiotics was identified.

Patients positive for airways MRSA were categorised as intermittently colonised or chronically infected according to a modification of the ‘Leeds Criteria’ defined for *P. aeruginosa* [10]. Briefly, intermittent MRSA colonisation was identified when ≤50 % of the months that respiratory samples were collected were culture positive (including patients who had one positive culture during the study period). Chronic MRSA infection was identified when >50 % of samples collected were culture positive.

As a subset analysis, longitudinal data for the MRSA positive HCWcf was collected to determine treatment outcomes. The first-line eradication regimen utilised at TPCH to treat MRSA infection of the CF airways was 6 months combination therapy of oral rifampicin 600 mg daily and sodium fusidate 500 mg twice daily after considering in vitro antibiotic susceptibility results [11]. Nasal and cutaneous decontamination was also undertaken if peripheral colonisation was detected. Eradication of MRSA, following 6 months of treatment was defined as six consecutive negative samples over a minimum period of 12 months.

### Data analysis

Demographics and clinical data were compared between groups using an independent *t*-test or Mann-Whitney test for continuous data and a Chi-squared test with Yates continuity correction or Fishers-Exact test for categorical data, as appropriate. Simple univariable and multivariable logistic regression analysis was performed to identify predictors of MRSA infection. Variables with *P* <0.1 were included in a multivariable model. Data analysis was performed using Stata (v14, StataCorp) or SPSS (v22). A *P* value of less than 0.05 was considered statistically significant.

## Results

### Patient characteristics for the entire cohort

During the study period, 21/405 (5 %) patients with CF worked in a healthcare discipline (Fig. 1). The vocations were comprised of medicine (*n* = 4); nursing/nursing assistant (*n* = 4); physiotherapist/assistant (*n* = 4); radiographer (*n* = 2); theatre attendant (*n* = 2); paramedic (*n* = 2); pharmacist (*n* = 1); phlebotomist (*n* = 1); small-animal veterinary surgeon (*n* = 1). All of the CF patients working in healthcare settings had frequent non-CF patient contact.

When we compared the demographic and clinical data between the non-HCWcf (*n* = 255) and HCWcf (*n* = 21), a statistical difference was only observed for lung function (Table 1). The FEV_1_ % predicted was higher among the HCWcf (median 72.1 % predicted) compared to the non-HCWcf (58.5 % predicted) [*P* = 0.003].

**Table 1:** Demographics and Clinical Characteristics of HCWcf and non-HCWcf

Characteristics	HCWcf		Non-HCWcf		*P* value
	No.^a^		No.^a^		
Age (years), median (IQR)	21	29 (23–37)	254	27 (21–34)	0.116
FEV_1_% predicted, median (IQR)	20	72.1 (61.3–90.9)	239	58.5 (37.9–73.1)	0.003
Number of hospitalisations in previous 2 years, median (IQR)	19	1 (0–3)	247	2 (0–5)	0.374
Number of hospital days, median (IQR)	19	9 (0–27)	246	16 (0–52)	0.251
Pancreatic insufficient, *n* (%)	21	16 (76)	254	221 (87)	0.177
*P. aeruginosa* infection, *n* (%)	20	15 (75)	255	224 (88)	0.157

^a^21 HCWcf and 255 non-HCWcf included in the analysis. Data missing on some occasions

### MRSA acquisition

Fifty patients with CF acquired MRSA (HCWcf, *n* = 10; non-HCWcf, *n* = 40, Fig. 1). A significantly higher proportion of HCWcf (*n* = 10/21, 48 %) acquired MRSA compared to non-HCWcf (*n* = 40/255, 16 %) (*P* <0.001). In a univariable logistic regression, the odds of MRSA acquisition were higher in the HCWcf compared to the non-HCWcf (Table 2). After adjusting for age and the number of hospital admissions in the previous 2 years (in a multivariable logistic regression) the odds of MRSA acquisition were 8.4 times higher in the HCWcf versus the non-HCWcf (Table 2).

**Table 2:** Regression analysis of factors associated with MRSA acquisition

Univariable	Odds ratio (95 % CI)	*P* value
Healthcare worker	4.87 (1.95–12.27)	0.001
Age (per 5 years)	0.79 (0.65–0.97)	0.026
FEV_1_% predicted (per 5 %)	0.98 (0.91–1.05)	0.514
Number of hospital admissions in previous 2 years (per admission)	1.11 (1.01–1.22)	0.028
Multivariable	Odds ratio (95 % CI)	*P* value
Healthcare worker	8.36 (2.99–23.39)	<0.001
Age (per 5 years)	0.78 (0.61–0.99)	0.039
Number of hospital admissions in previous 2 years (per admission)	1.13 (1.03–1.25)	0.011

Table 3 describes and compares the demographic, clinical and microbiological characteristics of both groups of MRSA positive patients (HCWcf versus non-HCWcf) at the time of acquisition. HCWcf were significantly older (*P* = 0.02) and had better lung function (FEV_1_ % predicted, *P* = 0.009) than non-HCWcf at the time of MRSA acquisition. Pancreatic sufficiency was more common within the HCWcf group compared to non-HCWcf (*P* = 0.048). There was no difference in sex (*P* = 0.494)*, P. aeruginosa* infection status (*P* = 0.086) or hospital days (*P* = 0.177) and admissions (*P* = 0.23) between the two groups. Furthermore, no difference in the acquisition of either mMRSA or nmMRSA was observed between the groups (*P* = 0.143).

**Table 3:** Demographics and Clinical Data of MRSA positive patients

Characteristics	HCWcf		Non-HCWcf		*P* value
	No.^a^		No.^a^		
Male, *n* (%)	10	5 (50)	40	25 (63)	0.494
Age (years), median (IQR)	10	28 (26–37.5)	39	22 (19–27)	0.02
FEV_1_ % predicted, median (IQR)	10	72.4 (62.2–90.9)	36	48.7 (33.2–64.8)	0.009
Pancreatic insufficient, *n* (%)	10	7 (70)	40	38 (95)	0.048
*P. aeruginosa* infection, *n* (%)	10	7 (70)	40	37 (93)	0.086
Hospitalisations in 2 years prior, median (IQR)	9	3 (1–4)	32	4 (2–6)	0.23
Hospital days in 2 years prior, median (IQR)	9	27 (4–37)	31	49 (19–76)	0.177
Peripheral colonisation, *n* (%)	9	2 (22)	33	9 (27)	1.0
mrMRSA, *n* (%)	10	5 (50)	40	30 (75)	0.143

*Abbreviations*: mrMRSA, multiresistant MRSA

^a^10 HCWcf and 40 non-HCWcf included in this analysis. Data missing on some occasions

### Chronicity of infection and treatment outcomes in a subset of individuals

Seventy-two percent of patients, who acquired MRSA (*n* = 36/50), subsequently developed chronic infection as defined by the Leeds Criteria. There was no statistical difference between rates of chronic MRSA infection when the two MRSA positive cohorts (HCWcf versus non-HCWcf) were compared (*P* = 0.44). Eleven of 42 (26 %) CF patients with MRSA airway isolation were also peripherally colonised with the bacterium (a documented skin swab result was not available for 8 patients). No association between colonisation of the skin by MRSA and HCWcf (22 %) or non-HCWcf (27 %) was detected (*P* = 1.0, Table 3).

All HCWcf with chronic MRSA respiratory infection (*n* = 6) received eradication therapy with successful eradication occurring in 5/6 (83 %) patients (Table 4). The remaining HCWcf (*n* = 4) had intermittent colonisation and cleared MRSA spontaneously.

**Table 4:** Types of infection and treatment outcomes among the 10 adult healthcare workers with CF (HCWcf) and methicillin-resistant *Staphylococcus aureus* (MRSA) airways infection

HCWcf (*n*)	Type of infection^a^	Antibiotic treatment^a^			Outcome		
		Rifampicin	Sodium fusidate	Linezolid	Eradicated	Chronic	Spontaneously cleared
1	Chronic	X	X		X		
2	Chronic	X	X		X		
3	Chronic	X	X		X		
4	Chronic	X	X		X		
5	Chronic	X	X			X	
6^b^	Chronic	X	X	X	X		
7^c^	Intermittent	X	X				X
8	Intermittent						X
9	Intermittent						X
10	Intermittent						X

^a^Median (IQR) time to initiation of treatment was 52 (37.5–77) days

^b^Patient was prescribed 6 weeks of linezolid following 6 months of rifampicin plus sodium fusidate

^c^Patient commenced eradication treatment but self-elected to cease after 3 weeks of therapy because of intolerance

## Discussion

The number of adults with CF is rapidly increasing with two thirds of this population in paid employment in Australia [12]. However, certain careers may increase the risk of exposure to harmful respiratory pathogens [13, 14]. It was also previously demonstrated that ~4.6 % of screened healthcare workers are colonised by MRSA [4]. In our study we present novel data investigating the association between working within a healthcare profession and the potential for MRSA acquisition in CF patients. We found that people with CF, who work in a healthcare profession, are at a greater risk of acquiring MRSA when compared to those who are not employed in this sector.

MRSA acquisition in people with CF differs both locally and internationally. For example, in Northern America the prevalence of MRSA exceeds 20 % of the total CF population [15], whereas in Australia, the point-prevalence in adults was reported as 4 % in 2013 [16]. Furthermore, at TPCH a decline in the annual prevalence of MRSA from 8.3 % in 2001 to 3.8 % in 2012 was observed (data not shown). It was previously reported that MRSA infection of the CF airways was associated with lower lung function in children and young people up to 21 years [7, 17], increased rates of hospitalisations and antibiotic requirements and worse survival compared to uninfected patients [6]. Therefore, it is important to identify and monitor MRSA infection in CF patients.

When the clinical characteristics were compared between HCWcf and non-HCWcf at the time of MRSA acquisition, it was observed that the HCWcf were healthier (indicated by better lung function) despite being older than the non-HCWcf. Whilst it is difficult to determine the reasons for better lung function of the HCWcf, the difference may reflect higher rates of pancreatic sufficiency, suggestive of a milder cohort of patients. Additionally, a healthier cohort may be attracted to study as healthcare professionals and undertake the rigors of gaining tertiary and/or professional qualifications.

Increased hospitalisations are a recognised risk factor for MRSA acquisition within a CF population [14]. There was no difference in the number of admissions or inpatient days between HCWcf and non-HCWcf with airways MRSA. Although cautious interpretation is required, we speculate that MRSA acquisition amongst the HCWcf may have occurred as a result of occupational exposure. However, it should be noted that our analyses did not extend to outpatient clinic or non-healthcare associated exposures and we cannot completely exclude MRSA acquisition during periods of hospitalisation; thus additional work is required to confirm our hypothesis.

Employment within a CF Centre theoretically poses one of the greatest risks to a HCWcf because of the strong body of evidence that has demonstrated the increased risk of cross-infection with other CF pathogens (including *P. aeruginosa*, *Burkholderia cepacia* complex and *Mycobacterium abscessus*) [14, 18–20]. However, an earlier case report of a HCWcf at TPCH (this patient is also included in the current study [patient #5, Table 4]) observed that MRSA respiratory acquisition was possibly the result of contact with non-CF patients at work [21]. Our study further highlights that healthcare employment, which involves frequent patient contact poses inherent risks to the person with CF. Previous studies demonstrated that MRSA is transmitted by direct person-to-person contact or via indirect contact with contaminated fomites [22, 23]. Furthermore, there is evidence indicating that Staphylococci may be capable of surviving in aerosolised particles within the respirable range [24, 25]. Studies are warranted to investigate the possibility of *S. aureus* transmission via the airborne route as was previously shown for *P. aeruginosa* [26].

There is a lack of evidence surrounding the role of eradication treatment of MRSA in CF and the most appropriate protocol to use [27]. In healthcare workers, it was also reported that decolonisation therapies varied but that eradication therapy was successful in 88 % of those treated [4]. At TPCH, factors used to determine the initiation of MRSA eradication treatment include chronicity of airways infection, potential adherence to the prolonged treatment regimen, clinical status and type of employment. In the current study, 72 % of CF patients developed chronic MRSA airways infection including 60 % of HCWcf. In non-CF healthcare workers, clinical infection with MRSA is infrequent with chronic infection developing occasionally (e.g. chronic MRSA sinusitis) [4, 28]. We have previously shown that prolonged treatment with rifampicin and sodium fusidate can eradicate chronic respiratory MRSA infection in a small number of adult CF patients [11] and in the current study 83 % of HCWcf achieved eradication with the same regimen. However, further prospective trials are needed to determine the best antibiotics to use, the duration of therapy and the effect on CF patient survival. It is also unclear if newly acquired MRSA infections should be treated in CF; however, preliminary results of the STAR-Too trial (NCT01349192) demonstrated that an eradication regimen for new MRSA airways colonisation was microbiologically efficacious and reduced pulmonary exacerbations in patients treated with combination oral and topical therapies coupled with environmental decontamination compared to a control observational group [29]. Evidence from our study indicated that only a quarter of CF patients with MRSA airways infection were also peripherally colonised with the bacterium. Therefore, larger studies are required for a risk-benefit analysis of topical decolonisation treatments (which can cause hypersensitivity reactions) in all patients with MRSA respiratory colonisation.

Limitations of this retrospective study include that a small number of patients were studied. However this reflects epidemiological data, which demonstrates that MRSA infection remains low in Australia compared to other recognised CF pathogens. Furthermore, annual acquisition of MRSA at TPCH has remained consistently low over the past decade (0–5 new cases per annum; incidence rate: 0–2.3 %; data not shown). Also, the results of this study were based on evidence collected from a single CF care centre in Australia. Although this centre cares for ~70 % of CF adults in Queensland, additional multi-centre studies, in other clinical settings where rates of MRSA endemicity vary, are required to confirm the findings of our study.

## Conclusions

To our knowledge this is the first study to demonstrate that acquisition of MRSA occurs more frequently amongst HCWcf than non-HCWcf. These data suggest that occupational exposure may increase the risk of MRSA acquisition for CF patients. Based on these findings we recommend that vocational guidance be provided to persons with CF who are considering pursuing a career in the healthcare industry. Likewise, there is an urgent need for the establishment of guidelines for CF Centres, healthcare training institutions and hospitals for the management of CF patients training and/or working in healthcare.

## Ethics approval and consent to participate

Ethics approval for this project was granted by The Prince Charles Hospital Human Research Ethics Committee, Metro North Hospital and Health Service, Brisbane, Queensland, Australia (HREC/13/QPCH/51). The Prince Charles Hospital Human and Research Ethics Committee approved that patient consent was not necessary for this retrospective observational study.

## Consent for publication

Not applicable.

## Availability of data and materials

The authors confirm that publication of the data does not compromise anonymity or confidentiality or breach local data protection laws. Data are available upon request.

## Abbreviations

CI: Confidence interval
FEV_1_%: Forced expiratory volume in one second percent
CF: Cystic fibrosis
HCWcf: Adult healthcare workers with CF
Non-HCWcf: Adult non-healthcare workers with CF
MRSA: methicillin-resistant *Staphylococcus aureus*
mMRSA: multiresistant MRSA
nmMRSA: non-multiresistant MRSA
TPCH: The Prince Charles Hospital
